# HIV and AIDS in Bangladesh

**DOI:** 10.3329/jhpn.v26i3.1898

**Published:** 2008-09

**Authors:** Tasnim Azim, Sharful Islam Khan, Fariha Haseen, Nafisa Lira Huq, Lars Henning, Md. Moshtaq Pervez, Mahbub Elahi Chowdhury, Isabelle Sarafian

**Affiliations:** ICDDR, B, Mohakhali, Dhaka 1212, Bangladesh

**Keywords:** Acquired immunodeficiency syndrome, HIV;, *Hijra*, Injecting drug user, MSM, Risk behaviours, Sex worker, Sexually transmitted infections, Bangladesh

## Abstract

Bangladesh initiated an early response to the HIV epidemic starting in the mid-1980s. Since then, the res-ponse has been enhanced considerably, and many HIV-prevention interventions among the most at-risk populations and the general youth are being undertaken. Alongside prevention activities, gathering of data has been a key activity fostered by both the Government and individual development partners. This paper reviews available sources of data, including routine surveillance (HIV and behavioural among most at-risk populations), general population surveys, and various research studies with the aim to understand the dynamics of the HIV epidemic in Bangladesh. Available data show that the HIV epidemic is still at relatively low levels and is concentrated mainly among injecting drug users (IDUs) in Dhaka city. In addition, when the passively-reported cases were analyzed, another population group that appears to be especially vulnerable is migrant workers who leave their families and travel abroad for work. However, all sources of data confirm that risk behaviours that make individuals vulnerable to HIV are high—this is apparent within most at-risk populations and the general population (adult males and youth males and females). Based on the current activities and the sources of data, modelling exercises of the future of the HIV epidemic in Dhaka suggest that, if interventions are not enhanced further, Bangladesh is likely to start with an IDU-driven epidemic, similar to other neighbouring countries, which will then move to other population groups, including sex workers, males who have sex with males, clients of sex workers, and ultimately their families. This review reiterates the often repeated message that if Bangladesh wants to be an example of how to avert an HIV epidemic, it needs to act now using evidence-based programming.

## HIV AND AIDS PROGRAMMES IN BANGLADESH

The first case of HIV in Bangladesh was detected in 1989. Even prior to this first case, the Government of Bangladesh (GoB) had become active and formed the National AIDS Committee (NAC) in 1985 in anticipation of an epidemic. The NAC is a high-profile body with the President as Chief Patron and Minister of Health and Family Welfare as the Chairperson. The NAC is supported by a Technical Committee and smaller subcommittees dealing with special issues that are formed when required. These subcommittees include the Surveillance Advisory Committee, the Estimates Working Group, and others.

The National AIDS/STD Programme (NASP), within the Directorate General of Health Services (DGHS) of the Ministry of Health and Family Welfare (MoHFW), acts as a nodal body responsible for programming to address HIV issues in the country. It was established to provide a high-level leadership from the Government and to facilitate programme implementation and coordination. The major roles of the NASP comprise policy, information, coordination and regulation, and implementation where necessary.

The GoB developed and approved a comprehensive policy on issues relating to HIV and AIDS and sexually transmitted infections (STIs) in 1997. The first National Strategic Plan (NSP, 1997–2002) and the second National Strategic Plan (2004–2010) have been developed and approved. The NSP provides the framework to guide response to the HIV epidemic. The priority areas are identified under five broad programme objectives, which include: provision of support and services for priority groups; prevention of vulnerability to HIV infection in Bangladesh society; promotion of safe practices in healthcare system; provision of care and support services for people living with HIV and AIDS; and minimizing the impact of the HIV and AIDS epidemic. The NASP also has been developing guidelines on antiretroviral treatment, harm-reduction strategy for drug-users, national STI management, national universal precaution protocol, voluntary counselling and testing (VCT), and training on safer sex strategy promotion.

To complement the efforts of the Government, a large number of NGOs are actively working on HIV and AIDS, primarily in prevention, and approximately 235 NGOs are interlinked through the STI/AIDS Network. Funding for all the HIV/STI activities has been and is obtained from the Ministry of Health and Family Welfare (MoHFW), GoB; the Global Fund for AIDS, TB and Malaria (GFATM); World Bank; DFID; German Technical Cooperation (GTZ); USAID; and others.

This paper reviews the existing situation of HIV in the country focusing on two main areas: state of the epidemic and issues around care and support.

## THE HIV EPIDEMIC: PRESENT STATUS AND THE FUTURE

**Fig. 1:**
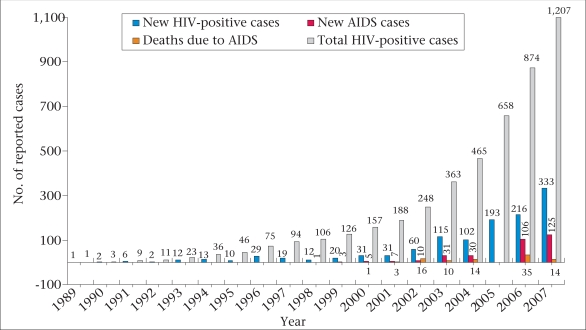
Number of reported HIV and AIDS cases, 1989–2007Year

The current estimates suggest an HIV prevalence rate of <1% among the most-at-risk population groups. The number of people living with HIV (PLHIV) and deaths from AIDS in Bangladesh are announced on the World AIDS Day every year by the MoHFW, These numbers have been steadily rising (Fig. 1). There were 1,207 reported HIV cases and 125 new AIDS cases at the end of 2007. Based on available data, the estimated number of infections in Bangladesh is about 7,500 (1).

**Surveillance data:** The national HIV surveillance system set up by the GoB has now been active since 1998. It is based on the UNAIDS/WHO guidelines for a revised ‘2^nd^ generation HIV surveillance’, a key priority of which is to improve the monitoring of developing epidemics like that in Bangladesh (2). The prevalence of HIV is monitored annually among specific groups at sentinel sites spread across the country. Behaviours that carry a risk of HIV infection are evaluated in tandem. Syphilis and hepatitis C (HCV) are also monitored as surrogate markers to corroborate behavioural data regarding unprotected sex and unsafe injections.

In accordance with the UNAIDS/WHO guidelines, HIV surveillance in Bangladesh has focused on selected groups of individuals known to be most-at-risk for acquiring HIV infection. They include sex workers, injecting drug users (IDUs), males who have sex with males (MSM), and *Hijra* (male transgenders). In addition, particular population subgroups, such as regular partners of sex workers or mobile men, including truckers and rickshaw-pullers, who may eventually be the source of spread of the epidemic into the general population are evaluated. In each of the past seven annual surveillance rounds, the pooled level of HIV prevalence was <1% among all population groups tested (3-14). However, the HIV epidemic in Bangladesh is evolving rapidly. While still a low-prevalence country for overall HIV rates, IDUs in Dhaka city have shown an increase of HIV prevalence from 1.4% to 7% in the past six years, and in one locality, this has now reached 10.5% (14). The surveillance data for each of the population groups will be discussed in the relevant sections.

### Injecting drug-users

**Fig. 2:**
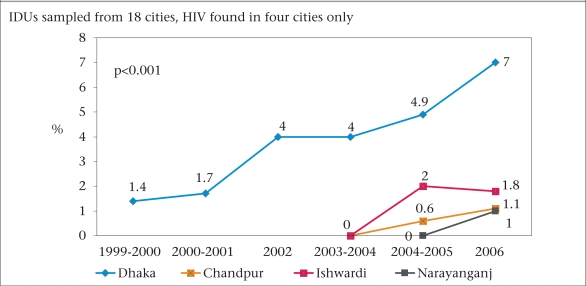
Prevalence of HIV among male IDUs: surveillance data71.11.84.91.7441.40.620010123456781999

IDUs are very vulnerable to an HIV epidemic, and this is the group in which the virus has been detected repeatedly. Data on IDUs are available from multiple sources, including the national surveillance, IDU research cohort studies (15,16), National Assessment of Situation and Responses to Opioid/Opiate Use in Bangladesh (17), and other Rapid Situation Response Assessments conducted by different NGOs. The surveillance system has documented a gradual rise in the prevalence of HIV in this group from Dhaka and in the 7^th^ Round, 2006, the prevalence rose to 7%. HIV was also detected, albeit in low levels, among IDUs from three other cities while no HIV was detected among IDUs from 14 of 18 cities (14) as shown in Figure 2.

In Dhaka, data from research and from the surveillance showed that the HIV epidemic was localized in a specific neighbourhood which could be considered to be the epicentre of the epidemic (14,18). Very high rates of HCV were seen among IDUs from most cities (3,4).

As IDUs are a group that was identified to be at the highest risk for an HIV epidemic, cohort studies were initiated among IDUs in Dhaka city and in Chandpur. Four cohorts of IDUs were: HIV-negative male IDUs in two neighbourhoods of Dhaka city (n=561); HIV-negative female IDUs (n=135) in Dhaka, Narayanganj, and Tongi; HIV-negative male and female IDUs in Chandpur (n=200); and an HIV-positive IDU cohort based in Dhaka (n=60). All studies were done in collaboration with an international NGO—CARE Bangladesh—which were conducting a needle/syringe programme (NSP) with IDUs. All persons in these IDU cohorts were followed biannually with assessment of risk behavi-ours, clinical examinations, and prevalence of HIV, HCV, and syphilis. Any person who was found to be HIV-positive became a member of the HIV-positive IDU cohort.

Recruitment for the male IDU cohort started in August 2002, and the baseline data for infection prevalence rates during January-March 2003 are shown in Table 1. At the end of the Dhaka male cohort study (in 2007), the incidence of HIV, HCV, and syphilis was, respectively, 1.29, 11.58, and 0.28 per 100 person-years. Although the incidence of HIV and syphilis is fortunately low, the high incidence rate of HCV is alarming and reflects the risky infection-sharing behaviour of IDUs.

**Table 1:** Baseline infection rates among a cohort of male IDUs in Dhaka

Type of infection	All IDUs (n=561)		Area A (n=361)		Area B (n=200)	
	No.	%	No.	%	No.	%
HIV	33	5.9	29	8.0	4	2.0
HCV	375	66.8	259	71.7	116	58
Active syphilis	19	3.4	15	4.2	4	2.0

HCV=Hepatitis C virus; HIV=Human immunodeficiency virus; IDUs=Injecting drug users

### Risk behaviours—injection practices

All sources of data on risk behaviours confirmed very risky injection-sharing behaviours among IDUs sampled from different cities. The national behavioural surveillance survey (BSS), conducted in 2003–2004 (12), showed that more than three-quarters of IDUs from Dhaka either borrowed or lent needles/syringes during the last injection. The average size of the sharing network for those IDUs who shared the last time varied from 1.0 to 2.8. Similar data were obtained from the male cohort study in Dhaka.

Qualitative data from the cohort study revealed that the concept of sharing needles/syringes among IDUs varied. Some believed that sharing with a family member or someone who appeared healthy did not amount to sharing while others believed that jerking the needle/syringe between sharing partners lowered the risk of infection through sharing.

In the cohort at the baseline, 48.3% of IDUs obtained their needles/syringes from the NSP and drug stores. The most common reason cited for sharing despite being in the NSP was not having access to sterile needles at the time of injection (57.8%) (16).

### Sexual behaviour of IDUs

In addition to risky injection practices, data showed that IDUs were also practising unsafe sex. In the cohort studies on male IDUs, approximately 50% were married, 36% had sex with their non-commercial partners (including wives), and 8% had bought sex from sex workers in the previous month. Of those buying sex, 51.2% had used condoms all the time in the last month (16).

### Female IDUs

Women who use drugs are known to be further marginalized and stigmatized and, as a result, are more vulnerable to HIV. Very little is known about female IDUs in Bangladesh, and intervention programmes are not accessing them in large numbers. We, therefore, attempted to set up a cohort of female IDUs from three cities—Dhaka, Tongi (27 km to the north of Dhaka city), and Narayanganj (23 km to the southeast of Dhaka city). All women aged 15 years and more with a history of injecting drugs at least once in the last six months were eligible for enrollment, and they were accessed with the help of outreach workers from the NSP of CARE Bangladesh, through the networks of female and male drug-users and that of female sex workers. During December 2004–March 2005, 135 female IDUs were enrolled in a cohort study, and the same study provided data for surveillance.

**Fig. 3:**
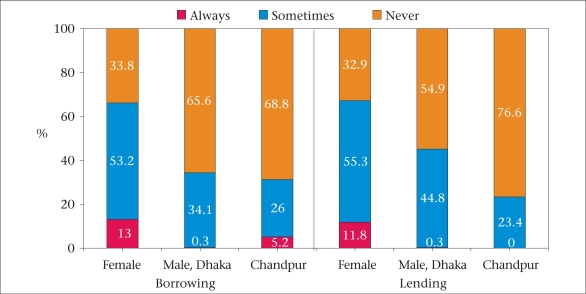
Frequency of sharing needles/syringes in the last week

At the baseline, none had HIV but 16.5% were infected with HCV, and 9.1% had syphilis. In the next survey, conducted six months later, one female IDU was detected with HIV. The rate of syphilis among female IDUs was higher than among male IDUs and similar to that of female sex workers. When injection risk-behaviours were compared for female IDUs with male IDUs in the different cohorts, female IDUs reported even higher needle-sharing rates than men (Fig. 3). The majority (63.1%) of female IDUs also reported selling sex in the last year, suggesting that this group is able to link the injection and sexual networks (15).

### HIV-positive IDUs

In 2007, 80 HIV-positive IDUs were identified—79 males and one female. Of them, 60 were married but only 28 were living with their spouses.

Attempts were made to identify the injection-sharing network of the HIV-positive IDUs by asking them who they had shared their injections with and where their partners could be found. Through this network, 96 sharing partners who were not already members of the cohort were identified and enrolled. Of them, 11.2% were HIV-positive. This rate is higher than the overall rate of HIV prevalence at the baseline of the IDU cohort where the total HIV prevalence was 5.9% (Table 1). Networking is a very effective way of reaching IDUs and may be considered a strategy for interventions as well.

### Are interventions working with IDUs? Modelling to assess the impact of intervention on IDUs

Despite all the risk-behaviours documented, the prevalence of HIV is still low in Bangladesh, and IDUs in Dhaka are the only group with an HIV epidemic. However, the rise in HIV prevalence is slow. To explore whether these better-than-expected results are related to the intervention activities, a mathematical modelling was conducted to estimate the impact of the NSP of CARE Bangladesh in Dhaka (19). Data from the NSP were used for parameterising a dynamic mathematical model and for fitting it to HIV serological surveillance data (2000–2002) among IDUs. The model was then used for estimating the impact of intervention on HIV transmission among IDUs and their sexual partners. The model predicts that the intervention may have reduced the incidence of HIV among susceptible IDUs by 89.5%, resulting in an HIV prevalence of 10.4% after eight years of intervention activity, instead of 42.0% if the intervention had not occurred. It is known that an effective NSP can reduce HIV transmission among IDUs and safer behaviours have been documented in IDUs who have participated in the Bangladesh NSP (20).

## SEXUAL TRANSMISSION OF HIV AND SEXUALLY TRANSMITTED INFECTIONS

### Female sex workers

Except for some small clusters of sex workers, the prevalence of HIV has remained <1% for female sex workers in all the rounds of surveillance. Surveillance for risk-behaviour has, however, shown that there is much cause for concern. Condoms were rarely used—usually less than 15% reported. High-risk behaviours, including anal sex, and group sex were also common. Somewhat encouraging has been some increased use of condoms, at least in some groups (12). Also, there has been a reduction in syphilis rates in female sex workers over time (14).

A qualitative study was conducted in 2003 to explore the patterns of condom-use among a sample of hotel-based female sex workers and their male clients who claimed to have used a condom during the last commercial sex act (21). Three specific patterns of condom-use were identified. Some clients started intercourse without a condom, but put one on before ejaculation, some started intercourse with a condom, but took it off after a few minutes or just before ejaculation, and others started intercourse with a condom and continued until the end. These findings question whether simply promoting condoms will effectively prevent the transmission of HIV.

Several separate surveys on STIs have been conducted with different groups of female sex workers. All showed high levels of different STIs, many of which were asymptomatic (22-25). These are summarized in Table 2.

**Table 2:** Sexually transmitted infections in female sex workers

Sexually transmitted infections	Percentage positive			
	Brothels (22)	Brothels (23)	Hotels (24)	Streets (25)
Cervical				
*N. gonorrhoeae*	11.5	17.5	35.8	35.6
*C. trachomatis*	13.2	15.5	43.5	25
Vaginal				
Ever syphilis (TPHA+, RPR <1:8)	Not done	31.5	8.5	32.6
Active syphilis (TPHA+, RPR >1:8)	5.7	6.6	4.2	Not done
*T. vaginalis*	35	7.5	4.3	45.5
HSV2	Not done	Not done	34.5	62.5

With these high rates of STIs, one wonders if the interventions of the NGOs are reducing risks. But these NGOs do appear to be making a difference as the behavioural surveillance data comparing sex workers in and out of interventions showed that those sex workers in interventions were practising safer behaviours (26).

### Male sex workers and *Hijra*

**Fig. 4:**
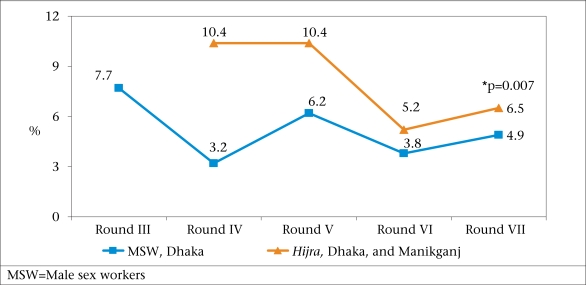
Active syphilis in male sex workers and *Hijra* over the round

Behavioural Surveillance Survey data have consistently shown that risk-behaviours in both male sex workers and *Hijra* are very high. Consistent condom-use in anal sex in the last week with new or regular clients was less than 25%. *Hijra* reported a very high average number of clients (30 in the last week). Condom-use was not common. Despite risky behaviours, HIV rates have remained <1% for both *Hijra* and male sex workers. Active syphilis rates remained similar in male sex workers but declined in *Hijra* over the rounds although the rates in *Hijra* were higher (Fig. 4).

A qualitative assessment of male-to-male sex was conducted in Chittagong in 2001, and one of the aspects explored was why men sell sex. The study showed that most men who sell sex, similar to most women who sell sex, did so for economic reasons. A common voice supported this assumption:

“I am not happy with my selling sex *pesha* (occupation). I feel pain during sex. I actually do not enjoy sex with men. I also face various types of violence. I cannot disclose my sex sell *pesha* to anyone in my family or society. I know this is an extremely bad behaviour. The Almighty Allah will punish me. I have no education. Who will give me job? I am a poor man; I cannot do any business either. How can I survive? I tried other daily labour to earn a living. But it is very difficult to survive. I found that selling my own body is a much better way to earn money. Every night I earn at least 100-200 taka. No one knows my source of income. I keep it hidden.”

### Males who have sex with males

The term ‘MSM’ has been used for categorizing males who have sex with males but do not necessarily identify themselves as ‘gay’ or ‘homosexual’. A survey of men in this category was undertaken to better understand these behaviours and attitudes. The survey revealed that these men did not call themselves ‘MSM’, nor did they like the terminolo-gy; in fact, they were annoyed by the term. Nonetheless, since this practice is known to be very risky for viral transmission, the term ‘MSM’ is used in Bangladesh. For the behaviour surveillance, the MSM group is defined as males who have sex with other males but do not sell sex. A recent study using a new sampling methodology, Respondent Driven Sampling (RDS), which is deigned to obtain a random sample from hidden populations, revealed that MSM in Dhaka are highly networked (27).

Although there were differences within the various geographic regions of the country, many of these men were also purchasing sex from males or *Hijra*. Group sex was reportedly common. Condoms were almost never used during sex. A large proportion of MSM had female sex partners or was married. A qualitative study attempted to understand the nature of the relations of MSM with women (28). It was found that these men feel societal pressure to marry, become husbands, and become fathers. Since these men were engaging in risky practices, their female partners were also at high risk for HIV and STIs (12).

### Mobile men

In 1998, a situational assessment of the Chittagong port was conducted to gather information on the behavioural factors contributing to the risk of HIV infection in the port city of Chittagong, Bangladesh, for the immediate objective of designing an HIV and STD-prevention programme (29). The populations included men from various trades in Chittagong and male and female sex workers. The study demon-strated an extensive clandestine sex trade in Chittagong. Traffickers brought women from Burma and the tribal areas of Bangladesh. Child prostitution was also common. Fishermen, dock-workers, and rickshaw-pullers represented major client groups, followed by local and foreign sailors and truckers. Between 40% (truckers) and 75% (dock-workers) of men reported recent contact with sex workers.

This study formed the basis of assessing the vulnerabilities of mobile populations in the surveillance system of Bangladesh through which dock-workers, truckers, rickshaw-pullers, and launch-workers were sampled in different rounds from different cities. HIV has not been detected in any of these groups sampled over the different rounds of surveillance with the exception of one rickshaw-puller out of 401 sampled in Dhaka during the 5^th^ Round of the serological surveillance (2003–2004)(12). However, high-risk behaviours for HIV/STIs have been recorded in the surveillance, and other special studies for truckers (30), rickshaw-pullers, and more recently in boatmen working in Teknaf (31). The latter study was conducted with boatmen who were aged ≥18 years living in Teknaf and working on boat-sailing from Teknaf in the last six months for trade, fishing, or carrying goods or passengers. Consistent condom-use among truckers and rickshaw-pullers in the last month with different partner types ranged from 1% to 11%. For boatmen, this was assessed over the last month, and it ranged from 0% to 4.7%.

### General population: males

Clients of sex workers are largely derived from the general community of men. Although men who stay away from their homes for prolonged periods (mobile men) may be practising more risky behaviours which make them more vulnerable to HIV/STIs, as discussed above, the other so-called general males are also very vulnerable. The differences in the reported behaviours in various studies (Table 3) are a reflection of the differences in the population groups that have been accessed and the study designs. However, the overall message is: at least 10% of men are buying sex from female sex workers.

**Table 3:** Proportion of men who reported sex with different types of sex partners (other than wives) in the last year in different studies conducted by ICDDR, B

Category of sex partner	Adult males 6 divisions* (n=7,122) Male sexual health survey (32)	Adult males (married) 6 divisions* (n=4,876) Male sexual health survey (32)	Adult males (married)† (n=407) Migration study (33)	University/college residential students, Dhaka‡ (n=339) 4th round BSS (13)
Female sex workers (%)	9.8	8.6	15.2	32.7
Female casual partners (%)	8.4	5.3	Not done	Not done
Males/*Hijra* (%)	2.1	0.8	2.5	4.5

*Adult males, aged 18-49 years, were enrolled from six divisions of Bangladesh—three urban (Dhaka metropolitan, Chittagong metropolitan, and Bogra town), and three rural areas (Faridpur, Rajshahi, and Cox's Bazar districts). The study was conducted during September 2004–August 2005

†Adult males, aged 15-49 years, were enrolled from Mirsarai and Abhoynagar. The study was conducted during October–December 2004

‡Adult male university/college students residing in dormitories. The surveillance was conducted in 2002; BSS=Behavioural surveillance survey

#### Low condom-use

Low condom-use by men (Table 4) remains one of the largest barriers to prevention of HIV in Bangladesh. Of the studies presented above, two on adult males showed a positive association between knowledge about prevention of HIV and condom-use (32,33). However, there are other more complex factors that need to be better understood for developing effective prevention programmes.

**Table 4:** Proportion of men who reported using a condom during the last sex with different types of sex partners (other than wives) in the last year in different studies conducted by ICDDR, B

Categories of sex partner	Adult males (all) 6 divisions*	Adult males (married) 6 divisions*	Adult males (married)†	University/college residential students, Dhaka‡
	Male sexual health survey (32)	Male sexual health survey (32)	Migration study (33)	4th round BSS (13)
Female sex workers (%)	40.1	31.5	6.5	35.3
Female casual partners (%)	30.0	Not analyzed	Not done	Not done
Males/*Hijra* (%)	8.7	7.2	0	18.8

*Adult males, aged 18-49 years, were enrolled from six divisions of Bangladesh—three urban (Dhaka metropolitan, Chittagong metropolitan, and Bogra town) and three rural areas (Faridpur, Rajshahi, and Cox's Bazar districts). The study was conducted during September 2004–August 2005

†Adult males, aged 15-49 years, were enrolled from Mirsarai and Abhoynagar. The study was conducted during October-December 2004

‡Adult male university/college students residing in dormitories. The surveillance was conducted in 2002; BSS=Behavioural surveillance survey

Qualitative studies among men revealed some deep-rooted issues that can act as barriers to condom-use (34). They revealed that direct penile-vaginal contact and ejaculation inside the vagina is the way men express their emotion and trust as they consider this to be a ‘pure’ and ‘natural’ sex act. This is exemplified by the quote below:

“Initially I began to use condoms, and both of us found that sexual interactions with condoms did not match our emotional intimacy. We do not have any barrier in our emotions and love, why should we place a ‘barricade’ in our sexual actions and emotions?”

By avoiding condoms, men sought to preserve an image of a ‘good man’, as condoms are equated with promiscuity.

“If I use condoms, this may indicate that I am concerned about *jouno rog* (STI) which would suggest that either one of us may be promiscuous. The meaning of love and trust will disappear.”

“I look unmarried. And you know how badly our society looks upon an unmarried man buying a condom. Being unmarried, how can I ask for condoms from a shopkeeper? I will be labelled a ‘bad boy’, who wants that image?”

### STIs in the general population (male and female)

Several studies have been conducted to assess the prevalence of STIs among men and women of the urban and rural general populations. Fortunately, rates of STIs have been very low in the general population (Table 5).

**Table 5:** Rates of sexually transmitted infection in the general population

Sexually transmitted infections	Data from studies conducted among different populations						
	Bogaerts (35) 1996–1998	Hawkes (36) 1995		Sabin (37) 1996		Bogaerts (38) 1996–1998	Hawkes (39) 1997
	Female, Dhaka	Male, Matlab	Female (married), Matlab	Male, Dhaka	Female, Dhaka		
*N. gonorrhoeae*	0.5	Not done	0.5	1.5	1.8	0.5	0.2
*C. trachomatis*	1.9	0.5	0.5	0.2	0.9	1.9	0.9
Ever syphilis (TPHA+, RPR-)	Not done	Not done	Not done	2.2	1.1	2.9	Not done
Active syphilis (TPHA+, RPR+)	Not done	0.5	0.7	9.3	4.4	Not done	0.9
*T. vaginalis*	2.0	0.8	Not done	Not done	Not done	2.0	1.5
HSV2	Not done	5.6	6.0	Not done	Not done	12.0	Not done

### Adolescents and youths

Several studies have been conducted among adolescent groups. One study was carried out to determine the effectiveness of a school-based intervention that combined the distribution of educational booklets and sensitization of key players, i.e. parents, teachers, community leaders, and service providers, to the need of providing adolescent reproductive health education. Its effect was measured in terms of changes in knowledge and practices regarding reproductive and sexual health as reported through pre- and post-intervention surveys. Students aged 13-19 years were assigned to one of the three groups depending upon what school they attended. Group A received community sensitization, booklet distribution, and training of providers in clinics, Group B received community sensitization and booklet distribution, and Group C served as controls.

The results demonstrated that the school-based intervention led to a significant increase in knowledge about STDs among adolescents but not specific knowledge about HIV and AIDS. Overall, the data suggest that there exist other sources of information, especially mass media that are also helping improve knowledge regarding HIV and AIDS. These findings support the conclusion that it will be difficult to attribute changes in knowledge or behaviour to a single intervention. Adolescents are being exposed to multiple sources of information, which make the assessment of changes extremely complex.

The study did provide valuable information on the process of implementing a culturally-sensitive intervention. Although the community recognized that reproductive health education is an important need, the efforts required in sensitizing the community indicate that reproductive health of adolescents remains a sensitive issue and will face many barriers if not sensitively planned and implemented.

Another community-based study assessed the reproductive health needs of adolescents aged 10-19 years. The survey data showed that most urban adolescent boys and girls had heard of AIDS, although there were differences between knowledge of slum and non-slum residents. Rural adolescents had, in general, significantly less knowledge about AIDS compared to that of urban adolescents. About half of the adolescents were ignorant of the routes of transmission of AIDS. About 90% of both rural and urban adolescents mentioned TV and radio as the prime sources of information regarding AIDS. About 15% of boys mentioned friends as a source of information on AIDS (15%).

## MIGRATION, GENDER, AND STIGMA

There are some special issues that are closely related to the HIV epidemic, including issues of migration, gender, and stigma. Although certain most at-risk populations have been the focus in the active surveillance, most cases identified through clinics have, in fact, acquired HIV while working abroad, or from their spouse who worked abroad (40). To date, 371 people attending the ICDDR, B's VCT Unit (Jagori) have been identified as infected with the virus. Of them, 54.2% are returnee-migrants, suggesting that migrants working away from their families may be particularly vulnerable to HIV.

Migration for work abroad is very common in Bangladesh. In two ICDDR, B surveillance areas in Mirsarai (Chittagong district) and Abhoynagar (Jessore district), 1,200 (10.8%) of married women of reproductive age have a husband living abroad. Return from working in a high-prevalence country is one of the ways HIV is introduced into low-prevalence countries. Similarly, a recent study in Nepal found that a high proportion of men working abroad had sex with a sex worker and had higher rates of HIV infection than those who had not left Nepal (41).

**Fig. 5:**
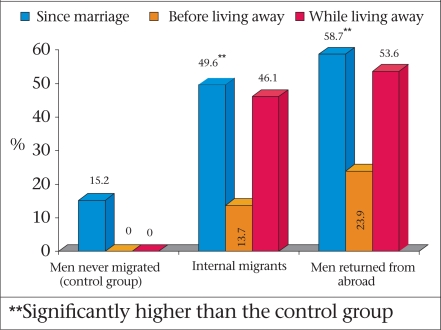
Proportion of men who had sex with a female sex worker

Reaching returnee-migrants for surveillance is difficult because they become part of the general population and do not form a special group. While they have special needs, targeting them might lead to stigmatization. Also, there was uncertainty as to their behaviours while abroad and when they return. A study was, therefore, undertaken among married couples to ascertain whether migration enhanced the vulnerability to HIV in both men and women (33,42). This was a cross-sectional survey of knowledge, behaviour, and practice among married men and women conducted in two ICDDR, B surveillance areas. The surveillance data allowed identification and sampling of subgroups of married men and women who had not lived apart from their spouses, were currently living apart, or had lived apart in the last five years. The findings showed that a large proportion of married men who had travelled either within Bangladesh or abroad reported sex with female sex workers (Fig. 5). A few men used a condom during sex with a sex worker (24-31%), or with their spouse (28-31%).

**Fig. 6:**
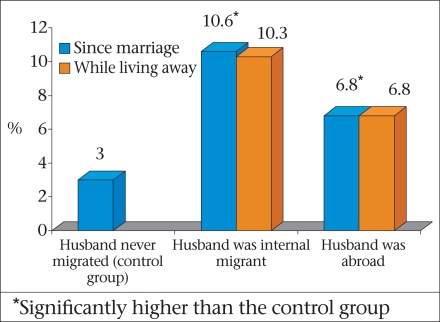
Proportion of married women who reported extramarital sex

Although not nearly as frequent, women whose husbands were away also reported more extramarital sex compared to those women who were not separated (Fig. 6). There is, thus, a clear evidence that migrants from Bangladesh are, indeed, vulnerable to HIV; however, there is still no strategy for reducing risk for migrants and their spouses without inducing stigma.

HIV and AIDS is also a gender issue since women are frequently the victims of HIV due to gender inequi-ty and male dominance. Bringing men into the discourse on HIV prevention has been a focus of global HIV-prevention efforts. To do this more effectively, understanding male sexuality and masculinity is necessary. Qualitative studies at ICDDR, B have shown that, for men in Bangladesh, ‘manhood’ is constructed by patriarchal norms, and relationships with women are within this construct so that men are men only when they can be ‘providers’ and ‘protectors’ of women (43). The sense of masculine responsibilities also creates sexual double standards and undermines sexual rights and equality of women in relationships. How to include concepts of gender in HIV-prevention programmes needs further research.

**Fig. 7:**
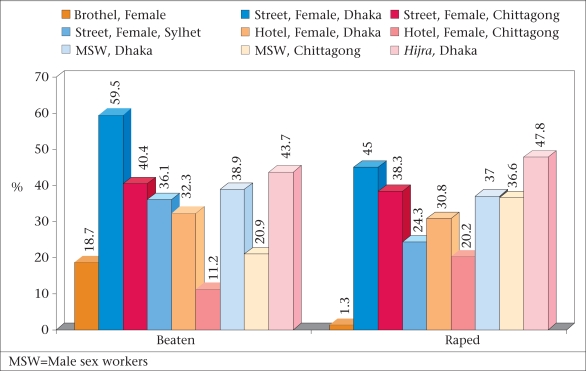
Violence against sex workers and *Hijra* (12)

Violence is also a major concern for the most-at-risk populations, particularly sex workers. Data on violence in different groups of sex workers (female, male, and *Hijra*) showed that nearly all groups are subject to violence (12), with street-based female sex workers and *Hijra* being the most affected (Fig. 7).

## THE FUTURE OF THE EPIDEMIC

Based on available data, modelling exercises have been conducted by Amala Reddy, Family Health International and Tim Brown, East-West Centre, Hawaii (44), using the Asian Epidemic Model (AEM) to predict the future course of the HIV epidemic in Dhaka. According to this model, the national epidemic is initiated by a localized epidemic in IDUs, and the epidemic increases explosively in this group to finally stabilize at about 70%.

This explosive growth of HIV prevalence among IDUs will then ‘seed’ the epidemic of sex work. Following or during the epidemic among sex workers, the prevalence of HIV will grow in other most at-risk-populations, such as MSM.

The virus will then continue its spread to the general population. Although the proportion will not reach the high levels like IDUs, the absolute numbers will be very high.

These models are only applicable for Dhaka, since this is the source of most data. They do not take into consideration the factors, such as the effect of migration, unsafe injections (in settings other than for IDUs), untested blood for transfusion, and others. However, despite these shortcomings with the available data, it is apparent that urgent and effective programming is essential.

## CARE AND SUPPORT SERVICES FOR HIV PATIENTS

There are limited care and support provisions for PLHIV in Bangladesh. Antiretroviral drugs (ARVs) are not available through the public healthcare systems yet. At present, the GoB focus on care for HIV and AIDS patients is primarily through NGOs, although a few dedicated doctors have taken this up on their own initiative. There is a considerable need for improving support from the medical establishments and improving the training to handle AIDS patients.

VCT services are the entry point for the provision of care and support for PLHIV. It is also an important service for providing preventive counselling to those people who perceive themselves to be at risk. In Bangladesh, VCT units have recently expanded and are run by different organizations, mainly NGOs. ICDDR, B has been running VCT units called Jagori in three locations—Dhaka, Chittagong, and Sylhet. Over the years, the number of clients seeking VCT services has increased, and this facility has seen more than 4,000 clients in the last six years. Much of the work of the VCT is preventive counselling, but the other services include physical examinations, arranging clinical investigations as needed, management of opportunistic infections, referrals to other service providers, clinical follow-ups of antiretroviral therapy (ART), and providing consultation services to other hospitals. HIV tests and CD4 counts are performed through the VCT. Common diagnoses among patients include oral thrush, fever, diarrhoea, skin infections, pneumonia, and tuberculosis. In 2007, a project on Prevention of Parent to Child Transmission (PPTCT) has commenced through which ARVs will be provided to HIV-positive pregnant women and their infected and affected families.

Six different types of ARV drugs are currently produced by Beximco Pharmaceuticals Ltd. and Square Pharmaceuticals Ltd.—both are national pharmaceutical companies in Bangladesh. More preparations are required to allow administration in different combinations, but paediatric formulations and ARV formulations for pregnant mothers are not available.

Information from the HIV-positive support groups suggests that approximately 95 PLHIV are receiving ARVs from different sources. A major obstacle to the proper management of PLHIV is affordability—the ARVs available are all being provided through short-term projects; CD4 counts are expensive and not widely available (available only in Dhaka), treatment of opportunistic infections is expensive. Besides, very few trained clinicians are available. Although there is a national guideline for ART, adherence to protocol is not assured, and hospitals are reluctant to admit patients who are known to be HIV-positive.

## SUMMARY

There is much good news in relation to HIV for Bangladesh. The prevalence remains low, certainly lower than the neighbouring countries. There has been strong government support for surveillance—both serological surveillance and behavioural surveillance—and the Government and NGOs have used these data to build the national programmes. Major sources of funds at present include the GoB and GFATM; sources of smaller funds are also available from USAID, GTZ, and others. The GoB and NGOs are working together on HIV.

The bad news relates to the highly risky behaviour of large groups of people who put themselves and others at risk of infection. There are many deeply-held cultural norms regarding acceptable behaviours, reluctance to use condoms, and gender issues that are major constraints to reducing the risk of an epidemic. Although migration has been a major source of new infections into Bangladesh, there have been no successful interventions to addressing the needs of migrants which are both effective and culturally acceptable. We do believe that the epidemic among IDUs—which is now starting—will be the focus of the impending, more general epidemic, and it would seem that much more successful strategies are needed to reduce sharing of needles, possibly including provision of clean oral drugs to these drug-users. The current strategy of treating them as ‘criminals’ will not be effective in stopping the transmission and may even be facilitating it. Thus, research is needed on how to effectively deal with this group.

Although donor agencies have provided substantial funding for HIV, the disbursement of these funds has not been consistent, which has led to difficulty in long-term planning for prevention, care, and support activities. Governmental support is needed for providing care and logistic services, including ARVs and provision for CD4 counts when needed. At this early stage of the epidemic, appropriate ARV therapy may be effective in slowing the transmission and slowing the epidemic. Effective interventions are evidence-based, and research is essential to better understand the factors driving the epidemic, and in some cases, there may even be ‘protective factors’ that need to be explored and tried as effective intervention strategies.

## References

[1] (2008). Bangladesh. Ministry of Health and Family Welfare. Directorate General of Health Services. National AIDS/STD Programme. 2008 UNGASS country progress report—Bangladesh; reporting period: January 2006-December 2007.

[2] (2000). Joint United Nations Programme on HIV and AIDS. Guidelines for second generation HIV surveillance: the next dicade.

[3] (2000). Bangladesh. Ministry of Health and Family Welfare. Directorate General of Health Services. National AIDS/STD Programme. Report on the sero-surveillance and behavioural surveillance on STD and AIDS in Bangladesh, 1998–1999. Editors: MR Choudhury, N Islam, C Jenkins, T Azim, and AM Hossain.

[4] (2000). Bangladesh. Ministry of Health and Family Welfare. Directorate General of Health Services. National AIDS/STD Programme. Report on the second national expanded HIV surveillance, 1999–2000 Bangladesh.

[5] Azim T, Islam MN, Bogaerts J, Mian MA, Sarker MS, Fattah KR (2000). Prevalence of HIV and syphilis among high-risk groups in Bangladesh. AIDS.

[6] (2001). Bangladesh. Ministry of Health and Family Welfare. Directorate General of Health Services. National AIDS/STD Programme. HIV in Bangladesh: where is it going?.

[7] Azim T, Bogaerts J, Yirrell DL, Banerjea AC, Sarker MS, Ahmed G (2002). Injecting drug users in Bangladesh: prevalence of syphilis, hepatitis, HIV and HIV subtypes. AIDS.

[8] (2003). Bangladesh. Ministry of Health and Family Welfare. Directorate General of Health Services. National AIDS/STD Programme. HIV in Bangladesh: is time running out?.

[9] (2003). Bangladesh. Ministry of Health and Family Welfare. Directorate General of Health Services. National AIDS/STD Programme. National HIV serological surveillance, 2000–2001, Bangladesh.

[10] (2004). Bangladesh. Ministry of Health and Family Welfare. Directorate General of Health Services. National AIDS/STD Programme. National HIV serological and behavioural surveillance, 2002; Bangladesh: fourth round technical report.

[11] Azim T, Alam MS, Rahman M, Sarker MS, Ahmed G, Khan MR (2004). Impending concentrated HIV epidemic among injecting drug users in Central Bangladesh. Int J STD AIDS.

[12] (2005). Bangladesh. Ministry of Health and Family Welfare. Directorate General of Health Services. National AIDS/STD Programme. National HIV serological surveillance, 2004–2005: Bangladesh. 6th Round technical report. Prepared by Tasnim Azim, Mahmudur Rahman, Md. Shah Alam, Imtiaz Ashraf Chowdhury, Motiur Rahman, and Masud Reza.

[13] (2007). Bangladesh. Ministry of Health and Family Welfare. Directorate General of Health Services. National AIDS/STD Programme. National HIV serological surveillance, 2003–2004, Bangladesh: fifth round technical report. Prepared by Tasnim Azim, Md. Shah Alam, and Motiur Rahman..

[14] Azim T, Rahman M, Alam MS, Chowdhury IA, Khan R, Reza M (2008). Bangladesh moves from being a low- prevalence nation for HIV to one with a concentrated epidemic in injecting drug users. Int J STD AIDS.

[15] Azim T, Chowdhury EI, Reza M, Ahmed M, Uddin T, Khan R (2006). Vulnerability to HIV infection among sex worker and non-sex worker female injecting drug users in Dhaka, Bangladesh: evidence from the baseline survey of a cohort study. Harm Reduct J.

[16] Azim T, Chowdhury EI, Reza M, Faruque MO, Ahmed G, Khan R (2008). Prevalence of infections, HIV risk behaviors and factors associated with HIV infection among male injecting drug users attending a needle/syringe exchange program in Dhaka, Bangladesh. Substance Use Misuse.

[17] Panda S, Mallick PS, Karim MA, Sharifuzzaman M, Ahmed AHT, Baastsen P (2002). National assessment of situation and responses to opiod/opiate use in Bangladesh (NASROB).

[18] Azim T, Hussein N, Kelly R (2005). Effectiveness of harm reduction programmes for injecting drug users in Dhaka city. Harm Reduct J.

[19] Foss A, Watt CJ, Vickerman P, Azim T, Guinness L, Ahmed M (2007). Could the CARE-SHAKTI intervention for injecting drug users be maintaining the low HIV prevalence in Dhaka, Bangladesh?. Addiction.

[20] Jenkins C, Rahman H, Saidel T, Jana S, Hussain AM (2001). Measuring the impact of needle exchange programs among injecting drug users through the national behavioural surveillance in Bangladesh. AIDS Educ Prev.

[21] Khan SI, Hasan MAK, Bhuiya A, Hudson-Rodd NSS (2003). How safe is sex with condoms?: an in-depth investigation of the condom use pattern during the last sex act in an urban area of Bangladesh. Int J Men's Health.

[22] Chowdhury ME (2005). Baseline survey for the HIV and AIDS prevention project: brothel-based sex workers in Bangladesh.

[23] Nessa K, Waris SA, Alam A, Huq M, Nahar S, Chawdhury FA (2005). Sexually transmitted infections among brothel-based sex workers in Bangladesh: high prevalence of asymptomatic infection. Sex Transm Dis.

[24] Nessa K, Waris SA, Sultan Z, Monira S, Hossain M, Nahar S (2004). Epidemiology and etiology of sexually transmitted infection among hotel-based sex workers in Dhaka, Bangladesh. J Clin Microbiol.

[25] Rahman M, Alam A, Nessa K, Hossain A, Nahar S, Datta D (2000). Etiology of sexually transmitted infections among street-based female sex workers in Dhaka, Bangladesh. J Clin Microbiol.

[26] (2004). MAP report. AIDS in Asia: face the facts. A comprehensive analysis of the AIDS epidemic in Asia.

[27] Johnston LG, Khanam R, Reza M, Khan SI, Banu S, Alam MS (2007). The effectiveness of respondent driven sampling for recruiting males who have sex with males in Dhaka, Bangladesh. AIDS Behav.

[28] Khan SI, Hudson-Rodd N, Saggers S, Bhuiya A (2005). Men who have sex with men's sexual relations with women in Bangladesh (short report). Cult Health Sexualit.

[29] Jenkins C (1998). A situational assessment of the Chittagong Port for HIV/STD prevention.

[30] Gibney L, Saquib N, Metzger J (2003). Behavioral risk factors for STD/HIV transmission in Bangladesh's trucking industry. Soc Sci Med.

[31] Gazi R, Mercer A, Wansom T, Kabir H, Saha NC, Azim T (2008). An assessment of vulnerability to HIV infection of boatmen in Teknaf, Bangladesh. Conflict Health.

[32] Chowdhury ME, Anwar I, Alam A, Ahmed A, DasGupta S, Mridha MK (2006). Assessment of sexual behavior of men in Bangladesh: a methodological experiment.

[33] Khanam R, Mercer A, Gurley E, Uddin J, Kabir H, Saha NC (2006). Vulnerability to HIV and AIDS of migration-affected families.

[34] Khan SI, Hudson-Rodd N, Saggers S, Bhuiyan MI, Bhuiya A (2004). Safer sex or pleasurable sex? Rethinking condom use in the AIDS era. Sex Health.

[35] Bogaerts J, Ahmed J, Akhter N, Begum N, van Ranst M, Verhaegen J (1999). Sexually transmitted infections in a basic healthcare clinic in Dhaka, Bangladesh: syndromic management for cervicitis is not justified. Sex Transm Infect.

[36] Hawkes S, Morison L, Chakraborty J, Gausia K, Ahmed F, Islam SS (2002). Reproductive tract infections: prevalence and risk factors in rural Bangladesh. Bull World Health Organ.

[37] Sabin KM, Rahman M, Hawkes S, Ahsan K, Begum L, Black RE (2003). Sexually transmitted infections prevalence rates in slum communities of Dhaka, Bangladesh. Int J STD AIDS.

[38] Bogaerts J, Ahmed J, Akhter N, Begum N, Rahman M, Nahar S (2001). Sexually transmitted infections among married women in Dhaka, Bangladesh: unexpected high prevalence of herpes simplex type 2 infection. Sex Transm Infect.

[39] Hawkes S, Morison L, Foster S, Gausia K, Chakraborty J, Peeling RW (1999). Reproductive-tract infections in women in low-income, low-prevalence situations: assessment of syndromic management in Matlab, Bangladesh. Lancet.

[40] Zaidi A, Zahiruddin M, Pervez MM, Sarker MS, Khan MR, Azim T Profile of HIV positive clients attending a VCT unit in Bangladesh. (abstract): *In*: Abstracts of the 15th International AIDS Conference, Bangladesh.

[41] (2002). Family Health International. New Era, STD/AIDS Counselling and Training Services. HIV/STD prevalence and risk factors among migrant and non-migrant males of Achham district in far-western Nepal.

[42] Mercer A, Khanam R, Gurley E, Azim T (2007). Sexual risk behavior of married men and women in Bangladesh associated with husbands’ work migration and living apart. Sex Transm Dis.

[43] Khan SI (2004). Male sexuality and masculinity: implications for STIs/HIV and sexual health interventions in Bangladesh.

[44] Reddy A, Kelly R, Brown T (2008). The Asian epidemic model for Dhaka city 2006: technical report.

